# Quality and reliability of information about blood glucose monitoring on TikTok and Bilibili: A cross-sectional analysis

**DOI:** 10.1371/journal.pdig.0001639

**Published:** 2026-08-24

**Authors:** Nanxiang Zhang, Jiayuan Cui, Peixuan Liu, Meiling Sun, Wei Wang, Bin Xu, Wanli Kong

**Affiliations:** 1 Department of Urology, Beijing Anzhen Hospital, Capital Medical University, Beijing, P. R. China; 2 Department of Anesthesiology and Surgery, Beijing Anzhen Hospital, Capital Medical University, Beijing, P. R. China; Khomein University of Medical Sciences, IRAN, ISLAMIC REPUBLIC OF

## Abstract

Monitoring blood glucose is essential for diabetes management, and many patients use short-video platforms to learn capillary blood glucose testing. We evaluated whether previously reported concerns about online health information also apply to this narrower procedural task and compared high-ranking videos on TikTok/Douyin and Bilibili. We included 192 videos (TikTok, n = 100; Bilibili, n = 92) and extracted duration, engagement metrics, uploader type, and video category. Two trained nursing reviewers assessed general educational quality and reliability-related characteristics using the Global Quality Score (GQS) and modified DISCERN (mDISCERN). Between-platform comparisons used Mann–Whitney U tests or Pearson’s chi-square tests, with standardized Mann–Whitney effect sizes; Spearman correlations examined associations with quality scores. TikTok videos received more likes, platform-specific saving actions, comments, and shares and were shorter than Bilibili videos (all P < 0.001; r = 0.315–0.403). TikTok also had higher proportions of accounts classified as professional uploaders (63.0% vs 35.9%, P < 0.001) and popular-science videos (53.0% vs 32.6%, P = 0.004). However, GQS and mDISCERN scores did not differ significantly between platforms (GQS, P = 0.246; mDISCERN, P = 0.072). Coverage of four prespecified procedural elements was incomplete on both platforms, with guidance on first-drop discard, alcohol drying, and post-puncture pressing time appearing in only a minority of videos. TikTok achieved substantially greater engagement, but popularity did not indicate significantly better educational quality or reliability-related characteristics. Standardized, professionally developed procedural videos may improve online diabetes self-management guidance.

## Author summary

Many people with diabetes use short videos to learn practical skills such as finger-stick blood glucose testing. We reviewed 192 publicly available, high-ranking videos on TikTok and Bilibili to assess whether they provided clear, reliable, and complete guidance for capillary blood glucose monitoring. TikTok videos received substantially more likes, comments, shares, and platform-specific saving actions than Bilibili videos, and TikTok included a higher proportion of uploaders who publicly presented themselves as healthcare professionals. However, general quality and reliability-related scores were only moderate and did not differ significantly between platforms. Both platforms commonly omitted or inconsistently presented the prespecified procedural details, including guidance on first-drop discard, allowing alcohol to dry before puncture, and post-puncture pressing time. Our findings show that popularity does not necessarily indicate better educational quality and that short videos should not replace professional instruction. Future platform-level studies should evaluate whether verification labels, standardized content templates, and professionally developed nursing videos improve the usefulness and safety of online diabetes self-management guidance.

## Introduction

Diabetes is a chronic metabolic disorder characterized by hyperglycaemia resulting from impaired insulin secretion, insulin action, or both, and its development is shaped by a combination of genetic susceptibility and environmental factors [1]. Beyond pharmacotherapy, dietary modifications----such as the consumption of whole-wheat breads--have been shown to significantly reduce fasting blood glucose (FBS) and HbA1c in patients with type 2 diabetes [2], underscoring the need for integrated patient education that covers both monitoring techniques and lifestyle adjustments. According to statistics from the International Diabetes Federation (IDF), in 2021, approximately 541 million adults worldwide had impaired glucose tolerance, and 319 million adults had impaired fasting glucose, accounting for 10.6% and 6.2% of the global adult population, respectively. These numbers are projected to rise to 730 million and 441 million by 2045 [3]. Effective glycaemic management depends heavily on patient self-management, yet adherence to core self-care behaviours is often suboptimal, especially among older adults [4]. Capillary blood glucose monitoring using portable glucose meters is one of the most widely used approaches for day-to-day glycaemic assessment and supports treatment adjustment and lifestyle management [5–8]. With the rapid growth of short-video platforms, many people now seek procedural guidance from social media; however, the accuracy and completeness of such content remain uncertain [9–11].

In this study, we evaluated and compared the content, general educational quality, reliability-related characteristics, source characteristics, and audience engagement of short videos about blood glucose monitoring on TikTok and Bilibili. Unlike broader assessments of diabetes-related social media content, our analysis focused on capillary blood glucose testing, a routine self-management procedure in which several practical steps may affect measurement accuracy and interpretation. This focus allowed us to examine whether previously reported concerns about the variable quality of health information on short-video platforms are also present in procedural diabetes education.

## Methods

### Ethical considerations

This study utilized publicly available data from short-video platforms; therefore, institutional review board approval and informed consent were not required.

### Study design and data sources

We conducted a cross-sectional study to evaluate the educational quality and reliability-related characteristics of short videos about blood glucose monitoring on Douyin, the Chinese domestic short-video platform referred to as TikTok in this manuscript, and Bilibili. No eligibility restriction was imposed on video publication date. All searches and initial data capture were conducted during a single standardized session on September 18, 2025 (China Standard Time, UTC + 8), using a single desktop computer. The searches were performed in incognito/private browsing mode while logged out of both platforms, after clearing the browser cache and browsing history to reduce personalized recommendation bias. The exact clock time of the search session was not prospectively recorded. On each platform, the first 100 results returned by the default comprehensive ranking displayed by the platform were included as the initial dataset. This search and sampling strategy was consistent with previous cross-sectional evaluations of health-related short videos on Chinese social media platforms, in which standardized searches and top-ranked results returned by the default platform ranking were used to approximate user-facing content exposure [12,13]. Standardizing the device, browser mode, login status, cache, and browsing history was intended to reduce, but could not eliminate, personalization and temporal variation in platform rankings. Accordingly, the present study was designed as a cross-sectional capture of the first 100 results displayed under the specified conditions on September 18, 2025, rather than as an attempt to reproduce a stable or exhaustive ranking of all blood glucose monitoring videos over time.

### Data retrieval and eligibility criteria

The standardized search strategy incorporated commonly used Chinese terms for blood glucose measurement and nursing operation videos. The Chinese search strings used on both platforms were “血糖监测”, “血糖测量”, “测血糖”, “护理血糖操作”, “血糖检测流程”, “指尖血糖”, and “毛细血管血糖测量”. These terms corresponded to the following English concepts: blood glucose monitoring, blood glucose measurement, blood sugar testing, nursing blood glucose operation, blood glucose testing procedure, fingerstick glucose, and capillary glucose measurement.

Inclusion criteria:

(1) publicly accessible videos with complete metadata (uploader identity, publication date, duration, likes, comments, the platform-specific saving metric, and shares);
(2) videos in which at least one information channel—visual content, narration, subtitles, or on-screen text—allowed the blood glucose monitoring procedure to be identified and assessed;
(3) explicit demonstration or instruction of blood glucose measurement or nursing operation steps;

Exclusion criteria:

(1) videos unrelated to blood glucose monitoring (e.g., diabetes education without operational demonstration, dietary advice, or pharmacologic management only);
(2) overt self-promotion, advertisements, or institutional publicity;
(3) duplicate or re-uploaded videos;
(4) incomplete or inaccessible videos, or videos for which neither the visual content nor the narration, subtitles, or on-screen text allowed the blood glucose monitoring procedure to be identified and assessed.

Two trained reviewers independently screened all retrieved videos. Discrepancies were resolved through discussion or adjudication by a senior nursing educator.

### Data extraction and classification

For each eligible video, we extracted the following information: platform, URL, title, publication date, duration (seconds), uploader category, and engagement metrics, including likes, comments, shares, and the platform-specific saving metric, namely favorites on TikTok and collections on Bilibili. Uploaders were classified into two categories based on publicly available profile information, account descriptions, displayed professional titles, institutional affiliation, and video content. Professional individuals were defined as accounts publicly presenting themselves as nurses, physicians, or other healthcare professionals, whereas non-professional individuals included patients, caregivers, students, or laypersons. Because offline professional credentials and platform verification status could not be independently confirmed for all uploaders, this classification reflected publicly identifiable professional presentation rather than independently verified licensure. All data were organized in Microsoft Excel (Microsoft Corp., Redmond, WA, USA) for further analysis. Coverage of key procedural elements was coded as binary variables, indicating whether each element was present or absent in the video. Discarding the first drop of blood was coded separately for mention and actual demonstration. “Mention” was defined as verbal narration, subtitles, or on-screen text indicating that the first drop should be discarded, whereas “actual demonstration” was defined as visually showing the action of discarding the first drop before measurement. Waiting for alcohol to dry before puncture and post-puncture pressing-time guidance were analyzed as content-coverage variables rather than continuous duration variables, because most videos did not provide a standardized numeric duration. These four content elements were selected for quantitative coding because they could be consistently identified from the video content and were directly related to common procedural details in capillary blood glucose monitoring. Other recommended educational components, such as hand hygiene, test-strip handling, blood volume adequacy, and interpretation of implausible results, were discussed as broader practice-oriented recommendations rather than coded as separate quantitative variables in the present study.

### Video quality and reliability assessment

Information quality was evaluated using the 5-point Global Quality Score (GQS), with higher scores indicating better overall educational value [14]. Information reliability was assessed with a 5-item modified DISCERN (mDISCERN) checklist (score range, 0–5), adapted from the original DISCERN instrument for consumer health information [14,15]. Because GQS and mDISCERN are general instruments for assessing the educational quality and reliability of online health information rather than procedure-specific checklists, they were used to evaluate the overall quality, clarity, balance, and reliability-related features of the videos. They were not used as direct measures of technical correctness for capillary blood glucose testing. To complement these general scoring tools, we separately assessed the coverage of key procedural elements, including discarding the first drop of blood, waiting for alcohol to dry before puncture, and providing post-puncture pressing-time guidance, as described above. Two trained nursing reviewers independently scored each video using both instruments. Whenever the two primary reviewers assigned different total scores on either the GQS or mDISCERN, the discrepancy was discussed with a third reviewer, who was a senior nursing educator, until all three reviewers reached consensus. Inter-rater reliability between the two primary reviewers was assessed for both the GQS and mDISCERN using a two-way random-effects, absolute-agreement, single-measure intraclass correlation coefficient [ICC(2,1)] based on their original independently assigned scores before the consensus discussion. ICC values were interpreted according to the thresholds proposed by Koo and Li: < 0.50 indicating poor reliability, 0.50-0.75 moderate reliability, 0.75-0.90 good reliability, and >0.90 excellent reliability [16].

### Statistical analysis

All analyses were performed using R software (version 4.4.0). Continuous and ordinal variables were summarized as median and interquartile range (IQR), and categorical variables were summarized as counts and percentages. Given the discrete, highly right-skewed, and zero-inflated nature of the engagement metrics, nonparametric tests were used without prior normality testing. Between-platform comparisons for continuous or ordinal variables, including GQS and mDISCERN scores, were performed using the Mann-Whitney U test. Categorical variables were compared using Pearson's chi-square test without Yates’ continuity correction. For Mann-Whitney U tests, standardized effect sizes were calculated as r = |Z|/sqrt(N), where Z was the standardized test statistic and N was the total number of videos; larger absolute values indicate larger between-platform separation. Spearman's rank correlation coefficient (rho) was used to evaluate associations among engagement indicators, video duration, uploader characteristics, video category, content coverage, and quality scores. For correlation analyses, uploader type was coded as 0 for non-professional individuals and 1 for professional individuals, and video category was coded as 0 for personal sharing and 1 for popular science. Each procedural-content variable was coded as 0 for absent and 1 for present; the content coverage score was the sum of the four binary variables and ranged from 0 to 4. Correlation strength was defined as negligible (|rho| < 0.2), weak (0.2-0.4), moderate (0.4-0.6), strong (0.6-0.8), or very strong (>0.8). For visualization in Fig 2, video duration and engagement metrics were displayed on a log10(value + 1) scale to accommodate zero values and improve interpretability, whereas all statistical comparisons were conducted on the original untransformed data. A two-sided P value < 0.05 was considered statistically significant.

**Fig 1:**
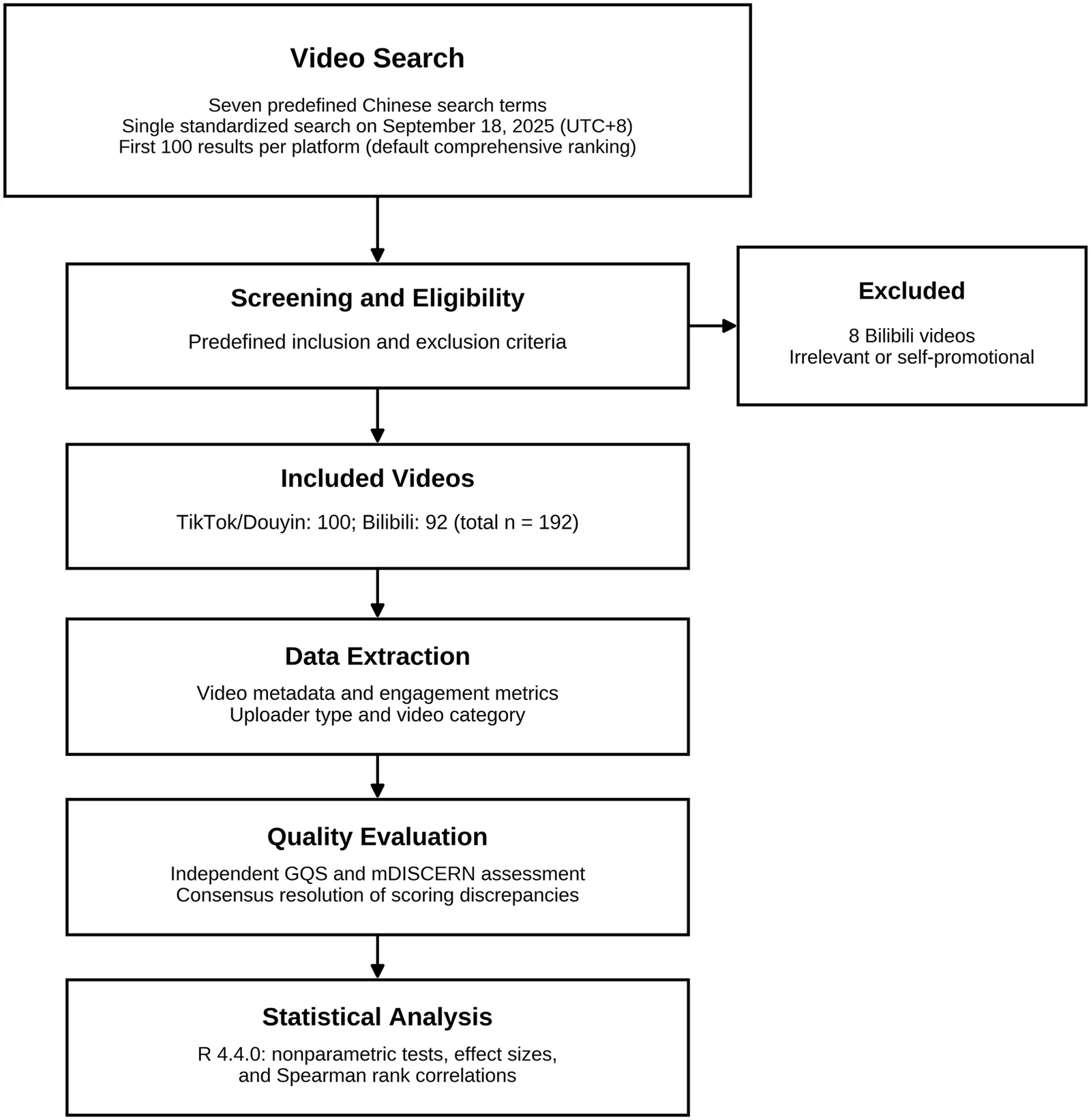
Flowchart of the selection process.

### Overview of the video selection process

The overall workflow of the study is illustrated in Fig 1 and comprised five stages: video search, screening and eligibility assessment, data extraction, quality evaluation, and statistical analysis. First, we selected two major platforms, TikTok/Douyin and Bilibili, and conducted all searches and initial data capture during a single standardized session on September 18, 2025 (China Standard Time, UTC + 8). The searches were performed in incognito/private browsing mode on a single desktop computer while logged out of both platforms, after clearing the browser cache and browsing history. No restriction was imposed on video publication date. On each platform, the first 100 results returned by the default comprehensive ranking were retrieved as the initial dataset. Second, duplicate and ineligible videos were removed according to the predefined criteria; eight Bilibili videos were excluded because they were irrelevant or self-promotional, leaving 100 TikTok videos and 92 Bilibili videos, for a total of 192 included videos. Third, we extracted video-level metadata, including the URL, publication date, duration, and engagement metrics, and classified uploader type and video category. Fourth, two trained nursing reviewers independently assessed each video using the GQS and mDISCERN scales. Any discrepancy between their independently assigned scores on either scale was discussed with a third reviewer, who was a senior nursing educator, until all three reviewers reached consensus. Finally, the data were analyzed in R software (version 4.4.0) using nonparametric tests, standardized Mann-Whitney effect sizes, and Spearman rank correlation analyses, with statistical significance defined as a two-sided P value < 0.05.

## Results

### Video selection

Ultimately, 100 TikTok videos and 92 Bilibili videos met the inclusion criteria and were included in the analysis, as shown in Fig 1.

### Descriptive characteristics of videos

**Fig 2:**
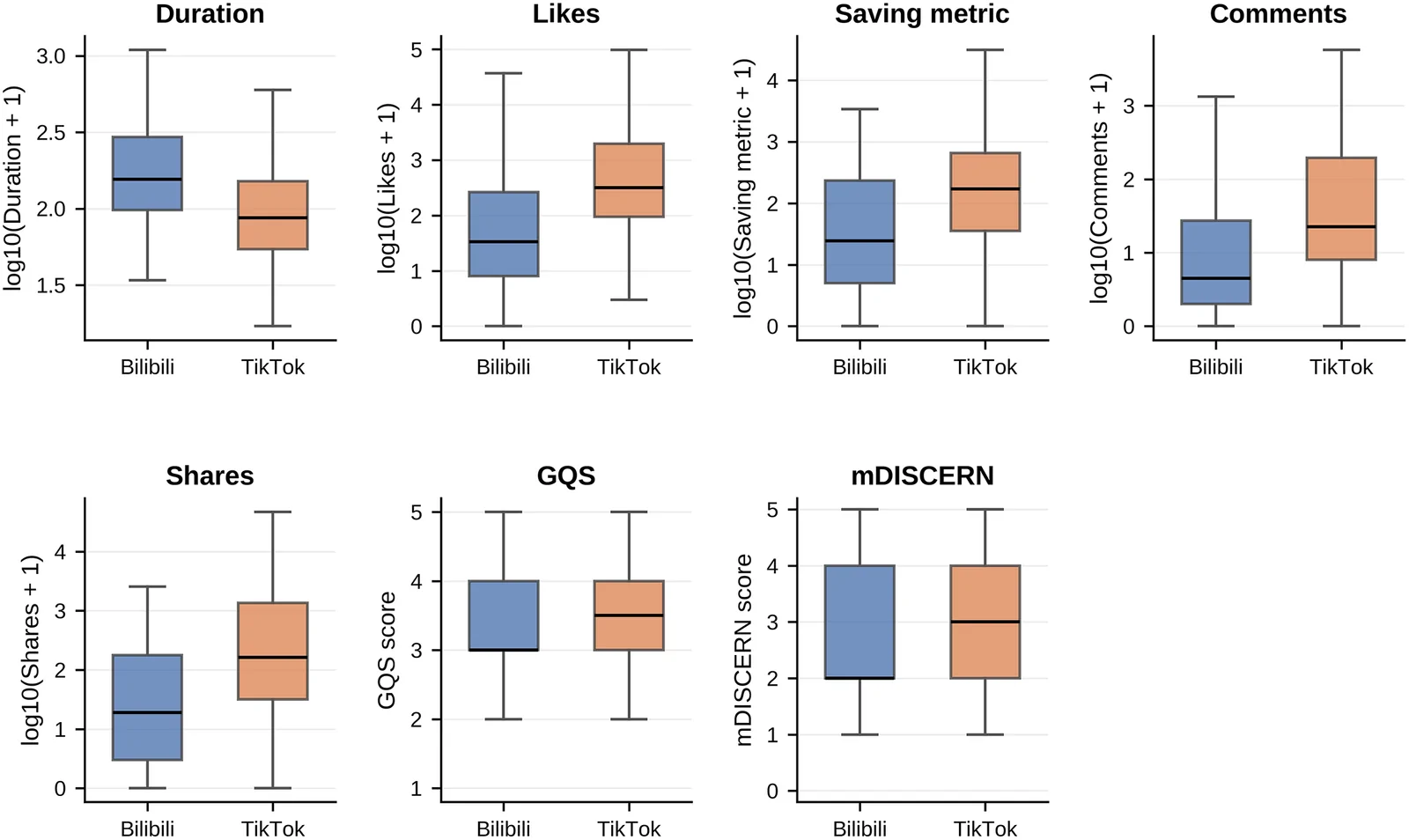
Comparison of video characteristics and engagement metrics between TikTok and Bilibili.

Descriptive characteristics of videos on TikTok and Bilibili are summarized in Fig 2 and Table A in S1 Appendix. Overall, TikTok videos demonstrated substantially higher audience engagement than Bilibili videos. For TikTok, the median (IQR) values for likes, the platform-specific saving metric (favorites), comments, and shares were 317.0 (93.25, 1961.50), 170.5 (34.50, 652.00), 21.5 (7.0, 194.0), and 162.5 (30.75, 1352.0), respectively. For Bilibili, the corresponding values, with collections representing the platform-specific saving metric, were 32.50 (7.00, 263.00), 23.50 (4.25, 232.00), 3.50 (1.00, 26.25), and 18.00 (2.00, 176.25), respectively. These medians corresponded to approximately 9.8-fold higher likes, 7.3-fold higher values for the platform-specific saving metric, 6.1-fold higher comments, and 9.0-fold higher shares on TikTok than on Bilibili. The standardized Mann-Whitney effect sizes were r = 0.403 for likes, r = 0.315 for the platform-specific saving metric, r = 0.354 for comments, r = 0.378 for shares, and r = 0.319 for duration. Statistical comparisons were performed on the original untransformed values. Inter-rater reliability was moderate for mDISCERN and excellent for GQS. Based on the two primary reviewers’ original independently assigned scores before the consensus discussion, the ICC(2,1) was 0.630 for mDISCERN and 0.950 for GQS. General educational quality and reliability-related scores were numerically higher on TikTok than on Bilibili, but the differences were not statistically significant (GQS, median [IQR]: 3.5 [3.0, 4.0] vs 3.0 [3.0, 4.0], P = 0.246, r = 0.084; mDISCERN, median [IQR]: 3.0 [2.0, 4.0] vs 2.0 [2.0, 4.0], P = 0.072, r = 0.130).

Boxplots show the median and interquartile range (IQR); whiskers extend to the most extreme observations within 1.5 times the IQR. Video duration and engagement metrics (likes, the platform-specific saving metric, comments, and shares) are displayed on a log10(value + 1) scale. GQS scores are shown on their original 1–5 scale, and mDISCERN scores on their original 0–5 scale. Colors indicate platform (Bilibili: blue; TikTok: orange).

### Characteristics of uploaders, video categories, and content features

**Fig 3:**
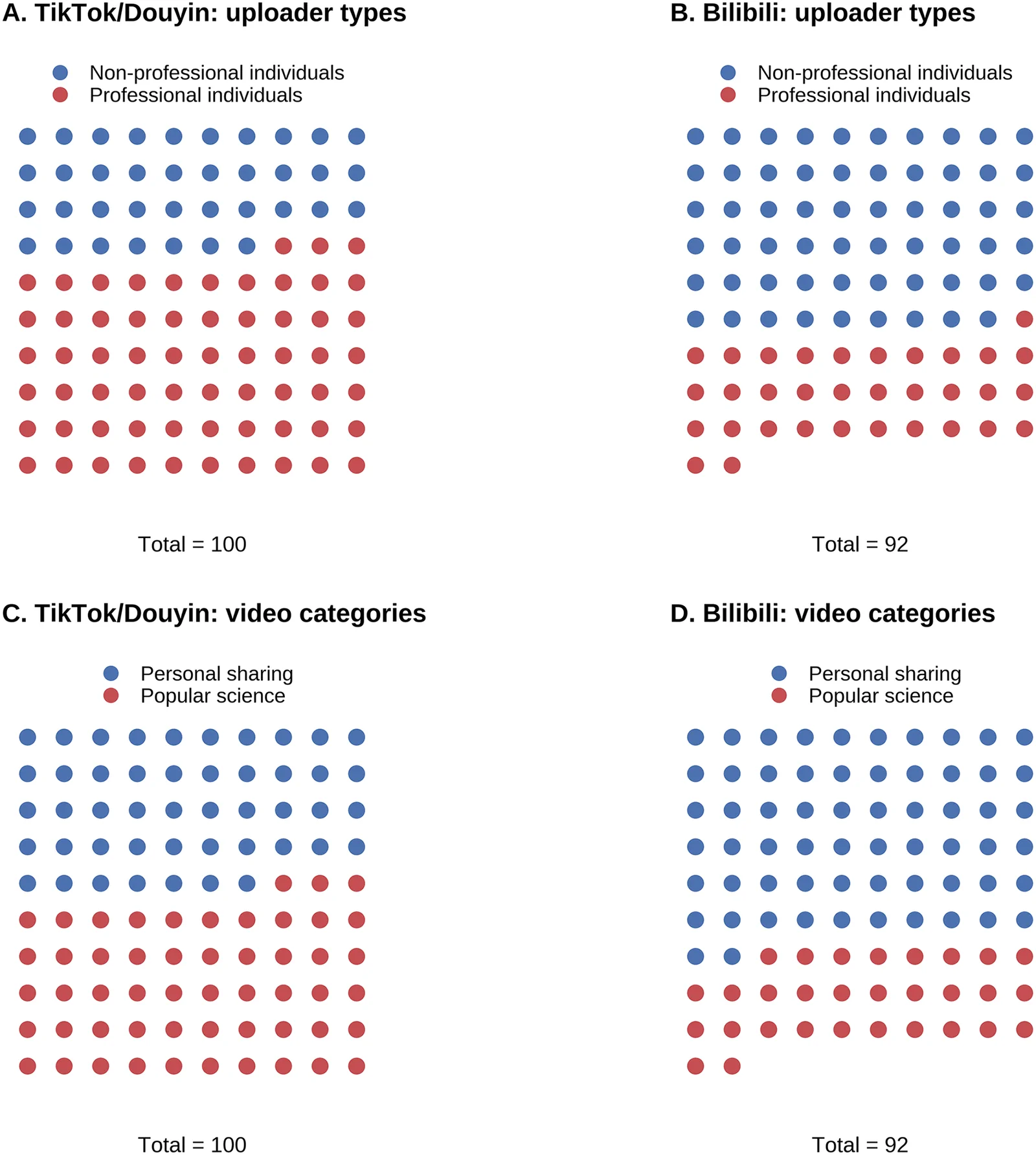
Distribution of uploader types and video categories across TikTok and Bilibili. (A) and (B) Waffle plots showing the proportions of videos uploaded by non-professional and professional individuals on TikTok and Bilibili, respectively. (C) and (D) Waffle plots displaying the proportions of personal case-sharing and popular-science videos on each platform. Each dot represents one video; total numbers of videos per platform are indicated below each panel.

As illustrated in Fig 3 and summarized in Table B in S1 Appendix, the composition of uploaders differed notably between platforms. On Bilibili, non-professional individuals accounted for 64.1% (59/92) of videos, whereas only 35.9% (33/92) were uploaded by professional individuals. In contrast, TikTok showed the opposite pattern: 63.0% (63/100) of videos were produced by professional uploaders and 37.0% (37/100) by non-professionals. These distributions show that TikTok had a higher proportion of accounts classified as professional uploaders, whereas Bilibili had a higher proportion of accounts classified as non-professional uploaders.

Regarding video categories, Bilibili was dominated by personal case-sharing videos, which represented 67.4% (62/92) of its content, whereas popular-science videos accounted for 32.6% (30/92). On TikTok, personal case videos comprised 47.0% (47/100) and popular-science videos 53.0% (53/100), suggesting a more balanced mix of experiential narratives and structured educational materials.

Both platforms showed incomplete coverage of the four prespecified procedural elements. Because most videos did not provide numeric durations for alcohol drying or post-puncture pressing, these variables were analyzed as binary presence-or-absence indicators rather than as continuous time variables.

**Fig 4:**
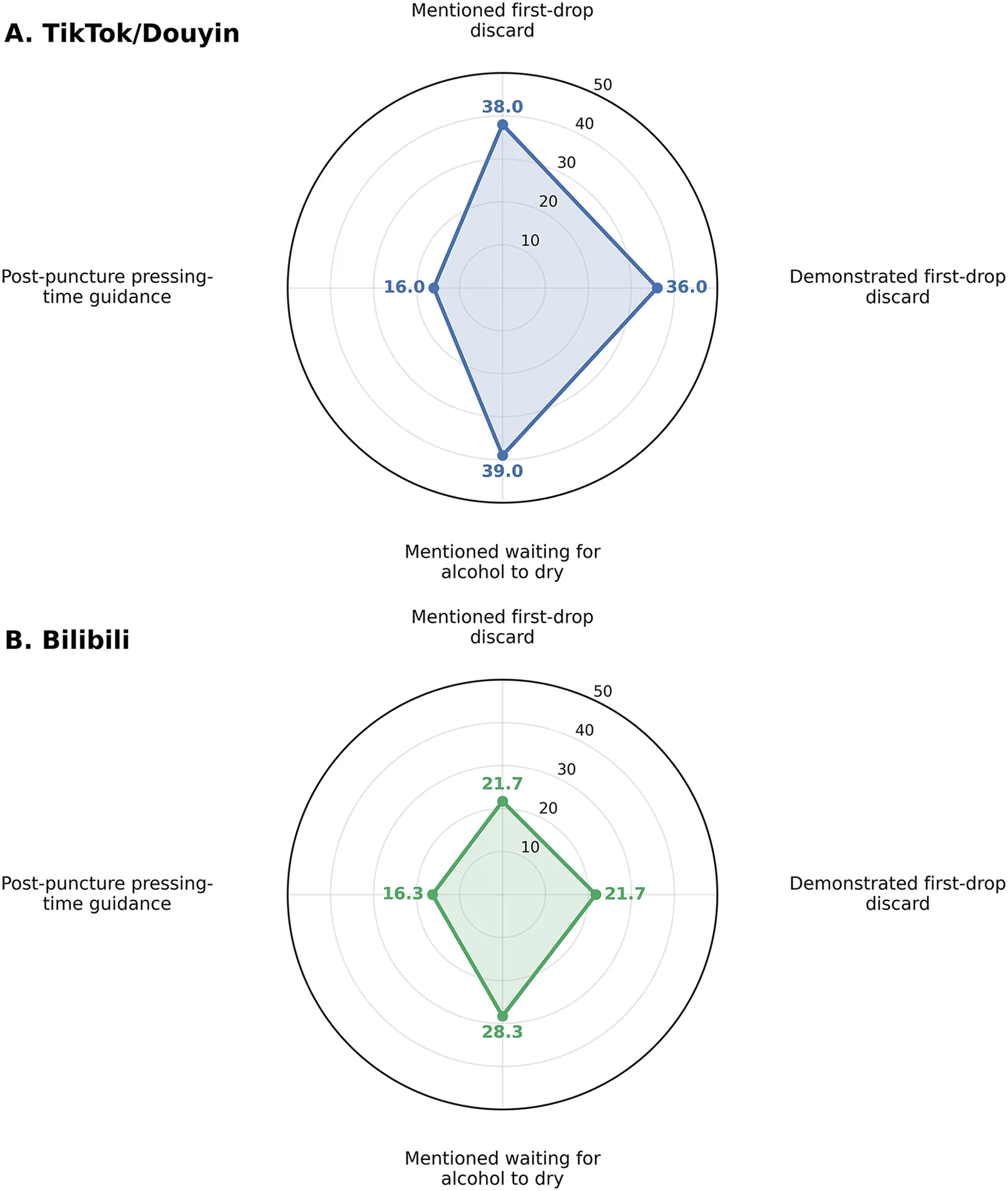
Coverage of key blood glucose monitoring content elements in TikTok and Bilibili videos. Radar charts show the percentages of TikTok and Bilibili videos that mentioned discarding the first drop of blood, demonstrated discarding the first drop, mentioned waiting for alcohol to dry, and provided post-puncture pressing-time guidance. Values are based on 100 TikTok videos and 92 Bilibili videos.

In addition to uploader and video category distributions, we examined coverage of four prespecified procedural elements (Fig 4 and Table D in S1 Appendix). On TikTok, 38.00% (38/100) of videos mentioned discarding the first drop of blood and 36.00% (36/100) demonstrated this step; the corresponding proportions on Bilibili were 21.74% (20/92) and 21.74% (20/92). Waiting for alcohol to dry before puncture was mentioned in 39.00% (39/100) of TikTok videos and 28.26% (26/92) of Bilibili videos. Post-puncture pressing-time guidance was uncommon on both platforms, occurring in 16.00% (16/100) of TikTok videos and 16.30% (15/92) of Bilibili videos. Thus, the observed proportions for first-drop mention, first-drop demonstration, and alcohol-drying guidance were descriptively higher on TikTok, whereas post-puncture pressing-time guidance was similarly uncommon on both platforms.

Spearman correlation analyses are presented in Table E in S1 Appendix. Engagement indicators showed weak associations with general educational quality and reliability-related scores: the platform-specific saving metric (rho = 0.215 with GQS, P = 0.0027; rho = 0.199 with mDISCERN, P = 0.0058) and shares (rho = 0.214 with GQS, P = 0.0028; rho = 0.192 with mDISCERN, P = 0.0078) were positively correlated with both scores. Video duration was also positively correlated with GQS (rho = 0.240, P = 0.0008) and mDISCERN (rho = 0.266, P = 0.0002). In contrast, comments were weakly and negatively correlated with GQS (rho = -0.161, P = 0.0262) and mDISCERN (rho = -0.183, P = 0.0109), whereas likes were not significantly correlated with either score (P > 0.05).

Uploader type, video category, and overall content coverage showed stronger positive correlations with both GQS and mDISCERN (uploader type: rho = 0.534 for GQS and 0.617 for mDISCERN; video category: rho = 0.617 for GQS and 0.631 for mDISCERN; content coverage score: rho = 0.691 for GQS and 0.710 for mDISCERN; all P < 0.001). These findings represent associations rather than causal effects. Videos from accounts classified as professional uploaders, videos classified as popular-science content, and videos covering more procedural elements tended to have higher scores, whereas raw popularity metrics showed only weak associations with the scores (Table E in S1 Appendix).

### Comparative analysis between platforms

As detailed in Table C in S1 Appendix and Fig 2, all four engagement metrics were significantly higher on TikTok than on Bilibili, whereas video duration was significantly shorter on TikTok (all P < 0.001). Standardized effect sizes ranged from r = 0.315 to r = 0.403. General educational quality and reliability-related scores were numerically higher on TikTok; however, no statistically significant between-platform differences were observed for either mDISCERN (P = 0.072; r = 0.130) or GQS (P = 0.246; r = 0.084). Significant differences were also found for uploader type (P < 0.001) and video category (P = 0.004): TikTok contained higher proportions of accounts classified as professional uploaders and popular-science videos, whereas Bilibili contained higher proportions of accounts classified as non-professional uploaders and personal sharing videos.

Overall, TikTok exhibited greater user engagement, shorter video duration, and higher proportions of accounts classified as professional uploaders and popular-science videos, whereas Bilibili featured longer videos and a higher proportion of personal, experience-based videos.

## Discussion

This study presents a comparative analysis of glucose monitoring-related videos on TikTok and Bilibili and identifies substantial between-platform differences in audience engagement and uploader characteristics. TikTok had higher viewer interaction and a higher proportion of accounts classified as professional uploaders. Although GQS and mDISCERN scores were numerically higher on TikTok, the between-platform differences were not statistically significant, indicating that general educational quality and reliability-related characteristics remained variable on both platforms.

TikTok included a higher proportion of accounts classified as professional uploaders and achieved higher engagement metrics across all four categories. The higher engagement observed on TikTok may reflect a combination of platform affordances, ranking and recommendation mechanisms, shorter-video format, and creator composition. However, because this study was cross-sectional and did not directly assess platform algorithms or user-level exposure, these factors should be interpreted as possible explanations rather than causal determinants. Bilibili contained a higher proportion of personal sharing videos and longer videos, but the present study did not test whether format or creator composition caused differences in engagement or quality. Similar platform-dependent disparities have been observed in prior studies evaluating health-related content on social media; broader reach on short-form platforms does not necessarily guarantee greater accuracy [9,11].

With the increasing number of people living with diabetes, precise digital communication has become crucial. Although self-monitoring of blood glucose is now routine for many individuals, patients may turn to social media rather than clinical consultation for procedural guidance. The variability in content quality across platforms underscores the need for professional input and evidence-based patient education materials. From a patient-education perspective, a high-quality instructional video should follow a standardized, step-by-step structure and explicitly address common sources of user error. However, health system interventions alone--such as Iran's Health Transformation Plan, which improved hospital performance metrics but failed to ensure educational continuity for outpatients [17]--demonstrate that structural changes must be paired with accessible, high-quality digital educational materials to achieve sustainable improvements in diabetes self-management.

The quantitative content analysis in this study assessed only the four prespecified elements reported in Fig 4 and Table D in S1 Appendix. To guide future content development, a broader practice-oriented framework informed by current guidelines can be organized into four domains. The first is preparation before sampling, including hand hygiene, appropriate disinfection, and waiting until alcohol has dried before puncture. The second is sampling technique, including selection of the lateral side of the fingertip, avoidance of excessive squeezing, and clear guidance on whether the first drop of blood should be discarded. The third is care after puncture, especially adequate compression of the puncture site and, when feasible, a clear recommended duration of pressure. The fourth is guidance on device use and result interpretation, including proper handling and storage of test strips, confirmation of sufficient blood volume, and advice to repeat testing or seek professional help when results are inconsistent with symptoms or otherwise implausible. These domains are consistent with general principles of capillary blood glucose monitoring and recent evidence regarding first-drop and second-drop sampling and could be evaluated as a practical checklist for future platform-level content templates [5–8,18,19]. Because the remaining elements in this broader framework were not separately coded in the present study, future research should quantify their coverage and evaluate their associations with educational quality and user outcomes. The potential effects of verification labels, standardized templates, and professional oversight should also be evaluated prospectively rather than assumed from the present findings.

Although TikTok videos generated substantially higher engagement (likes, comments, the platform-specific saving metric, and shares) and showed numerically higher GQS and mDISCERN scores, the differences in general educational quality and reliability-related characteristics between platforms were not statistically significant; importantly, both platforms showed incomplete coverage of the prespecified procedural elements. The low barrier to content creation on social media enables non-professionals to disseminate demonstrations of blood glucose monitoring without clinical oversight, and oversimplified or inaccurate presentations may lead patients to adopt suboptimal techniques and obtain inaccurate readings. Accordingly, professional nurses, diabetes educators, and medical associations should take a more active role in creating, reviewing, and disseminating standardized, patient-friendly videos that are accurate, balanced, and easy to follow [9,10,20–24].

The integration of social media into healthcare communication is an irreversible reality. Rather than dismissing TikTok or Bilibili as entertainment channels, they can be studied as potential extensions of patient education and public health outreach. Future platform-level studies should test whether verification labels, peer-reviewed digital media, and collaboration between clinical and communication specialists improve content quality, user comprehension, and safe self-management. Nonetheless, this study has several limitations. First, we analyzed only publicly accessible videos, and all searches and initial data capture were completed during a single standardized session on September 18, 2025 (China Standard Time, UTC + 8). Although the search environment was standardized by using a single desktop computer, incognito/private browsing mode, logged-out access to both platforms, and cleared browser cache and browsing history, these measures could not eliminate algorithmic and temporal variation in platform rankings. The exact clock time was not prospectively recorded, and the study did not include repeated independent searches at different time points; therefore, the exact ranked result set should not be expected to recur identically in a later search. Search rankings and engagement metrics may change because of platform algorithm updates, content changes, and temporal fluctuations in user interaction. Accordingly, the findings should be interpreted as a date-specific cross-sectional snapshot of the first 100 high-ranking results displayed under the specified conditions, rather than as a stable or exhaustive representation of all videos or all time periods. Second, cross-platform comparisons of engagement indicators should be interpreted cautiously because the platform-specific saving metric (favorites on TikTok and collections on Bilibili) may not represent identical user behaviors across the two platforms. Differences in user interface design, bookmarking visibility, recommendation algorithms, and cultural usage patterns may have confounded direct comparisons of audience engagement. In addition, uploader classification was based on publicly available account information and video content, and we could not independently verify professional licensure or platform verification status for all uploaders. Third, the scoring systems (mDISCERN and GQS) are susceptible to inter-rater variability, and differences in language use and presentation style between platforms may have influenced comprehension and scoring. In addition, although GQS and mDISCERN are widely used to assess online health information, they were not originally developed specifically for procedural blood glucose monitoring videos. Therefore, the scores in this study should be interpreted as indicators of general educational quality and reliability-related characteristics rather than as definitive measures of procedural accuracy. This limitation was partly addressed by separately evaluating the coverage of key procedure-specific content elements. Finally, because of the cross-sectional and observational nature of this study, associations among uploader type, video category, content coverage, engagement metrics, and quality scores should not be interpreted as evidence of causal relationships. Future research should incorporate longitudinal data and audience surveys to assess the real-world impact of these videos on patient understanding and decision-making.

In summary, TikTok videos on blood glucose monitoring achieved substantially higher audience engagement and included higher proportions of accounts classified as professional uploaders and popular-science videos than Bilibili videos, while general educational quality and reliability-related scores were moderate and did not differ significantly between platforms. There remains considerable room for improvement in the completeness, standardization, and clinical clarity of video content on both platforms. Encouraging the development and prospective evaluation of professionally supervised, standardized educational videos may help support safer and more informed self-monitoring practices.

## Supporting information

S1 Data Minimal dataset used in the analyses. This Excel file contains one row for each of the 192 included videos and all variables required to reproduce the descriptive statistics, figures, between-platform comparisons, standardized effect-size estimates, correlation analyses, content-coverage analyses, and inter-rater reliability estimates. The original GQS and mDISCERN scores independently assigned by both primary reviewers before the consensus discussion are included. A variable codebook and data notes are provided in the same file.
(XLSX)

S1 Appendix Supplementary tables. This appendix contains the supplementary tables referenced in the main text: Table A (descriptive statistics of engagement metrics and information quality scores of videos on TikTok and Bilibili), Table B (distribution of uploader types and video categories across TikTok and Bilibili), Table C (baseline characteristics, between-platform comparisons, and Mann–Whitney effect sizes), Table D (coverage of key blood glucose monitoring content elements), and Table E (Spearman correlations between video characteristics/engagement metrics and quality scores).
(DOCX)
