# Supplementary material for: Quality and reliability of information about blood glucose monitoring on TikTok and Bilibili: A cross-sectional analysis
Source: PLOS Digit Health. 2026 Aug 24;5(8):e0001639. doi: 10.1371/journal.pdig.0001639 (PMC13502678; doi:10.1371/journal.pdig.0001639)
Supplement: Supplementary file 1 [file pdig.0001639.s002.docx]

**Supplementary Tables**

This appendix contains the supplementary tables referenced in the main text (Tables A-E).

**Table A. Descriptive statistics of engagement metrics and information quality scores of videos on TikTok and Bilibili.**

| **Platform** | **Statistic** | **Duration** | **Likes** | **Saving metric** | **Comments** | **Shares** | **GQS** | **mDISCERN** |
| --- | --- | --- | --- | --- | --- | --- | --- | --- |
| Bilibili | N | 92.00 | 92.00 | 92.00 | 92.00 | 92.00 | 92.00 | 92.00 |
| Bilibili | Mean | 249.93 | 1095.93 | 272.17 | 107.76 | 220.84 | 3.28 | 2.67 |
| Bilibili | SD | 305.50 | 4277.72 | 610.62 | 384.58 | 499.07 | 0.89 | 1.24 |
| Bilibili | Min | 6.00 | 0.00 | 0.00 | 0.00 | 0.00 | 2.00 | 1.00 |
| Bilibili | Max | 2367.00 | 37000.00 | 3366.00 | 3114.00 | 2540.00 | 5.00 | 5.00 |
| Bilibili | Median | 155.00 | 32.50 | 23.50 | 3.50 | 18.00 | 3.00 | 2.00 |
| Bilibili | P25 | 97.00 | 7.00 | 4.25 | 1.00 | 2.00 | 3.00 | 2.00 |
| Bilibili | P75 | 293.50 | 263.00 | 232.00 | 26.25 | 176.25 | 4.00 | 4.00 |
| Bilibili | IQR | 196.50 | 256.00 | 227.75 | 25.25 | 174.25 | 1.00 | 2.00 |
| Bilibili | SE | 31.85 | 445.98 | 63.66 | 40.09 | 52.03 | 0.09 | 0.13 |
| Bilibili | 95% CI | 186.67-313.20 | 210.04-1981.83 | 145.72-398.63 | 28.12-187.40 | 117.48-324.19 | 3.10-3.47 | 2.42-2.93 |
| TikTok | N | 100.00 | 100.00 | 100.00 | 100.00 | 100.00 | 100.00 | 100.00 |
| TikTok | Mean | 134.51 | 3305.24 | 1245.57 | 337.06 | 1890.69 | 3.40 | 2.97 |
| TikTok | SD | 138.64 | 10826.50 | 4003.38 | 834.56 | 5826.70 | 0.93 | 1.18 |
| TikTok | Min | 5.00 | 2.00 | 0.00 | 0.00 | 0.00 | 1.00 | 1.00 |
| TikTok | Max | 779.00 | 97519.00 | 31116.00 | 5730.00 | 46745.00 | 5.00 | 5.00 |
| TikTok | Median | 86.00 | 317.00 | 170.50 | 21.50 | 162.50 | 3.50 | 3.00 |
| TikTok | P25 | 53.25 | 93.25 | 34.50 | 7.00 | 30.75 | 3.00 | 2.00 |
| TikTok | P75 | 150.25 | 1961.50 | 652.00 | 194.00 | 1352.00 | 4.00 | 4.00 |
| TikTok | IQR | 97.00 | 1868.25 | 617.50 | 187.00 | 1321.25 | 1.00 | 2.00 |
| TikTok | SE | 13.86 | 1082.65 | 400.34 | 83.46 | 582.67 | 0.09 | 0.12 |
| TikTok | 95% CI | 107.00-162.02 | 1157.03-5453.45 | 451.21-2039.93 | 171.46-502.66 | 734.55-3046.83 | 3.22-3.58 | 2.73-3.21 |

Abbreviations: CI, confidence interval; GQS, Global Quality Score; IQR, interquartile range; SD, standard deviation; SE, standard error.

Notes: Values are provided for descriptive completeness. Because engagement metrics were highly right-skewed and included zero values, the main text and between-platform comparisons primarily used median, interquartile range, and non-parametric tests. Mean, SD, SE, and 95% CI are presented as supplementary descriptive information only. Saving metric refers to favorites on TikTok and collections on Bilibili.

**Table B. Distribution of uploader types and video categories across TikTok and Bilibili.**

| **Video** | **Platform** | **Category** | **n (%)** |
| --- | --- | --- | --- |
| Uploader type | Bilibili | Non-Professional individuals | 59 (64.13%) |
| Uploader type | Bilibili | Professional individuals | 33 (35.87%) |
| Uploader type | TikTok | Non-Professional individuals | 37 (37.00%) |
| Uploader type | TikTok | Professional individuals | 63 (63.00%) |
| Video category | Bilibili | Personal sharing | 62 (67.39%) |
| Video category | Bilibili | Popular science | 30 (32.61%) |
| Video category | TikTok | Personal sharing | 47 (47.00%) |
| Video category | TikTok | Popular science | 53 (53.00%) |

Notes: Professional uploaders were identified based on publicly available profile information, account descriptions, displayed professional titles, institutional affiliation, and video content. This classification reflected publicly identifiable professional presentation rather than independently verified licensure or platform verification status.

**Table C. Baseline characteristics, between-platform comparisons, and Mann-Whitney effect sizes.**

| **Variables** | **Total (n=192)** | **Bilibili (n=92)** | **TikTok (n=100)** | **Statistic** | **Effect size r** | **P** |
| --- | --- | --- | --- | --- | --- | --- |
| Duration, M (Q1, Q3) | 120.00 (64.50, 230.75) | 155.00 (97.00, 293.50) | 86.00 (53.25, 150.25) | \|Z\| = 4.42 | 0.319 | <.001 |
| Likes, M (Q1, Q3) | 130.00 (25.00, 963.50) | 32.50 (7.00, 263.00) | 317.00 (93.25, 1961.50) | \|Z\| = 5.58 | 0.403 | <.001 |
| Saving metric, M (Q1, Q3) | 80.50 (12.00, 466.50) | 23.50 (4.25, 232.00) | 170.50 (34.50, 652.00) | \|Z\| = 4.36 | 0.315 | <.001 |
| Comments, M (Q1, Q3) | 13.50 (2.00, 102.25) | 3.50 (1.00, 26.25) | 21.50 (7.00, 194.00) | \|Z\| = 4.90 | 0.354 | <.001 |
| Shares, M (Q1, Q3) | 70.00 (9.00, 404.50) | 18.00 (2.00, 176.25) | 162.50 (30.75, 1352.00) | \|Z\| = 5.24 | 0.378 | <.001 |
| GQS, M (Q1, Q3) | 3.00 (3.00, 4.00) | 3.00 (3.00, 4.00) | 3.50 (3.00, 4.00) | \|Z\| = 1.16 | 0.084 | 0.246 |
| mDISCERN, M (Q1, Q3) | 3.00 (2.00, 4.00) | 2.00 (2.00, 4.00) | 3.00 (2.00, 4.00) | \|Z\| = 1.80 | 0.130 | 0.072 |
| Uploader type, n (%) |  |  |  | Pearson chi-square = 14.11 | -- | <.001 |
| Non-Professional individuals | 96 (50.00) | 59 (64.13) | 37 (37.00) |  |  |  |
| Professional individuals | 96 (50.00) | 33 (35.87) | 63 (63.00) |  |  |  |
| Video category, n (%) |  |  |  | Pearson chi-square = 8.12 | -- | 0.004 |
| Personal sharing | 109 (56.77) | 62 (67.39) | 47 (47.00) |  |  |  |
| Popular science | 83 (43.23) | 30 (32.61) | 53 (53.00) |  |  |  |

Abbreviations: GQS, Global Quality Score; M, median; Q1, first quartile; Q3, third quartile.

Notes: Continuous variables are presented as median (Q1, Q3). Categorical variables are presented as n (%). Absolute Z values from the Mann-Whitney U tests are reported because effect size r was calculated as |Z|/sqrt(N), where N was the total number of videos; the direction of each between-platform difference is indicated by the group medians. Categorical variables were compared using Pearson's chi-square test without Yates' continuity correction. Saving metric refers to favorites on TikTok and collections on Bilibili.

**Table D. Coverage of key blood glucose monitoring content elements.**

| **Video content element** | **Bilibili** | **TikTok** | **Total** |
| --- | --- | --- | --- |
| Mentioned discarding first drop | 20 (21.74%) | 38 (38.00%) | 58 (30.21%) |
| Performed discarding first drop | 20 (21.74%) | 36 (36.00%) | 56 (29.17%) |
| Mentioned waiting for alcohol to dry | 26 (28.26%) | 39 (39.00%) | 65 (33.85%) |
| Provided post-puncture pressing-time guidance | 15 (16.30%) | 16 (16.00%) | 31 (16.15%) |

Notes: Data are presented as n (%). Each content element was coded as present or absent. Alcohol-drying and pressing-time items were analyzed as binary content-coverage variables because most videos did not provide standardized numeric durations.

**Table E. Spearman correlations between video characteristics/engagement metrics and quality scores (GQS and mDISCERN).**

| **Video characteristic** | **Spearman rho (GQS)** | **P value (GQS)** | **Spearman rho (mDISCERN)** | **P value (mDISCERN)** |
| --- | --- | --- | --- | --- |
| Likes | 0.069 | 0.3405 | 0.052 | 0.4744 |
| Saving metric | 0.215 | 0.0027 | 0.199 | 0.0058 |
| Comments | -0.161 | 0.0262 | -0.183 | 0.0109 |
| Shares | 0.214 | 0.0028 | 0.192 | 0.0078 |
| Duration | 0.240 | 0.0008 | 0.266 | 0.0002 |
| Uploader type | 0.534 | P < 0.001 | 0.617 | P < 0.001 |
| Video category | 0.617 | P < 0.001 | 0.631 | P < 0.001 |
| Content coverage score | 0.691 | P < 0.001 | 0.710 | P < 0.001 |
| Mentioned discarding first drop | 0.516 | P < 0.001 | 0.529 | P < 0.001 |
| Performed discarding first drop | 0.478 | P < 0.001 | 0.510 | P < 0.001 |
| Mentioned waiting for alcohol to dry | 0.633 | P < 0.001 | 0.649 | P < 0.001 |
| Provided post-puncture pressing-time guidance | 0.447 | P < 0.001 | 0.499 | P < 0.001 |

Notes: Spearman rank correlation coefficients (rho) and corresponding P values are shown. Uploader type was coded as 0 for non-professional individuals and 1 for professional individuals; video category was coded as 0 for personal sharing and 1 for popular science. Content coverage score was calculated as the sum of the four binary content elements listed in Table D, with a possible range from 0 to 4; higher scores indicate more complete coverage of the prespecified procedural elements. Saving metric refers to favorites on TikTok and collections on Bilibili.
